# Impact of tailored blogs and content on usage of Web CIPHER – an online platform to help policymakers better engage with evidence from research

**DOI:** 10.1186/s12961-016-0157-5

**Published:** 2016-12-01

**Authors:** Steve R. Makkar, Megan Howe, Anna Williamson, Frances Gilham

**Affiliations:** The Sax Institute, Level 13, Building 10, 235 Jones Street, Ultimo, NSW 2007 Australia

**Keywords:** Health policy, Research, Websites, Portals, Innovations, Policymakers, Knowledge translation

## Abstract

**Background:**

There is a need to develop innovations that can help bridge the gap between research and policy. Web CIPHER is an online tool designed to help policymakers better engage with research in order to increase its use in health policymaking. The aim of the present study was to test interventions in order to increase policymakers’ usage of Web CIPHER. Namely, the impact of posting articles and blogs on topics relevant to the missions and scope of selected policy agencies in the Web CIPHER community.

**Methods:**

Five policy agencies were targeted for the intervention. Web CIPHER usage data was gathered over a 30-month period using Google Analytics. Time series analysis was used to evaluate whether publication of tailored articles and blogs led to significant changes in usage for all Web CIPHER members from policy agencies, including those from the five target agencies. We further evaluated whether these users showed greater increases in usage following publication of articles and blogs directly targeted at their agency, and if these effects were moderated by the blog author.

**Results:**

Web CIPHER usage gradually increased over time and was significantly predicted by the number of articles but not blogs that were posted throughout the study period. Publication of articles on sexual and reproductive health was followed by sustained increases in usage among all users, including users from the policy agency that targets this area. This effect of topic relevance did not occur for the four remaining target agencies. Finally, page views were higher for articles targeted at one’s agency compared to other agencies. This effect also occurred for blogs, particularly when the author was internal to one’s agency.

**Conclusion:**

The findings suggest that Web CIPHER usage in general was motivated by general interest, engagement and appeal, as opposed to the agency specificity of content and work relevance. Blogs in and of themselves may not be effective at promoting usage. Thus, in order to increase policymakers’ engagement with research through similar online platforms, a potentially effective approach would be to post abundant, frequently updated, engaging, interesting and widely appealing content irrespective of form.

**Electronic supplementary material:**

The online version of this article (doi:10.1186/s12961-016-0157-5) contains supplementary material, which is available to authorized users.

## Background

There has been growing emphasis on the importance of developing innovations that facilitate the transfer of research evidence into health policymaking [1–3]. A number of innovations have been developed and tested to support policymakers’ capacity to engage with and use research in policy [4, 5]. Some of these interventions utilise network- or web-based technologies due to the potential for such tools to reach a wide audience of decision-makers quickly and efficiently [6, 7]. Knowledge platforms are one such web-based technology that has been increasingly used to improve how information is accessed, stored, shared, retrieved and distributed within organisations [8, 9]. Evidence suggests that knowledge platforms could be used to overcome some of the barriers to Evidence Informed Health Policymaking such as difficulties in accessing, applying and disseminating up-to-date, credible and applicable research to policymakers. Furthermore, these platforms may be useful for organisations that lack tools and systems to support research access, generation and use in policy, as well as to establish and sustain policymakers’ partnerships with researchers [10–16].

The Centre for Informing Policy in Health with Evidence from Research (CIPHER), is a centre for research excellence located in New South Wales, Australia, which was established to develop and test innovative ways to increase the use of research in health policymaking. One of the innovations developed by CIPHER was Web CIPHER (http://cipher.org.au), an online web portal designed to help health decision-makers better utilise, access and engage with evidence from research, with the ultimate goal of increasing their use of research in their work. Upon its inception, Web CIPHER’s target audience was policymakers, practitioners and researchers from Australian agencies that routinely developed health policies, programs and practices, at either the state or federal level.

Web CIPHER utilises web and email technology to facilitate access to and delivery of relevant and up-to-date information about health research and policy. The website is password-protected and contains five key sections with regularly updated content: Hot Topics, Research Updates, Events, Multimedia and Blog. The site also includes static sections, such as the Research Portal and Research Tools, as well as an interactive Community Bulletin Board to facilitate communication between policymakers and researchers. These sections are described in Table 1.

**Table 1:** Details of the main features of each section of Web CIPHER

Section	Update frequency	Content	Selection criteria for content	How the section targets barriers to evidence-informed policymaking
Hot Topics	Daily	Provides summaries and blurbs of news articles that are directly relevant to member agencies of Web CIPHER; direct links to these articles are provided	Media monitoring with keywords of interest set up via Google Alerts (e.g. “public health”, “research”, “evidence”, and “health policy”); articles are scanned daily and chosen for their relevance and interest to members of the Web CIPHER network; the emphasis is on intervention research, program reviews, locally-relevant news, or examples of evidence-based policy	Many of the selected articles promote the value and importance of evidence-informed decision making in health
Research Updates	Fortnightly	Summaries of systematic reviews, research papers and reports, with links to full articles	Fortnightly scan of relevant journals and sources for systematic reviews, research articles and reports (e.g. *Australian and New Zealand Journal of Public Health*, *Implementation Science*); these sources are reviewed for research, reviews and reports of interest, with an emphasis on public health intervention research, program evaluation, locally-relevant research, and research translation	Articles are chosen specifically for their relevance to agencies within the Web CIPHER community; numerous articles focus on strategies to facilitate research use in policy; article summaries are written in an engaging, newspaper style to highlight the main findings, aid comprehension of full text articles, and highlight the policy relevance of findings
Events	Monthly	Summaries of upcoming events of interest to health policymakers	Monthly scan of organisations of interest for relevant events (e.g. the Australian Commission on Safety and Quality in Health Care, the NSW Agency for Clinical Innovation, the National Health & Medical Research Council, the Australian Research Council, the Cochrane Collaboration) and organisations represented by Web CIPHER members	The section allows policymakers to learn about upcoming programs, seminars, and conferences to improve their research skills, expose them to the latest research findings, or provide opportunities to connect with researchers
Multimedia	Monthly	Videos, audio recordings and presentations of interest to health policymakers	Monthly scan of email alerts from a range of organisations (e.g. the Australian Healthcare & Hospitals Association, the Australian Commission on Safety and Quality in Health Care) and organisations represented by Web CIPHER members	The media files posted in this section often discuss practical strategies and innovations to integrate research into policy, as well as the value of using research to inform policymaking
Blogs	Monthly-quarterly	500-word conversational-style articles written by health leaders, focusing on their area of expertise, with lessons for policymakers	Quarterly meetings with management team to agree on potential topics and contributors; pitch sent to contributor, and article written by contributor in consultation with senior staff member at the Sax Institute to ensure tone and style matches Web CIPHER requirements	Blogs are often geared towards improving policymakers’ perceptions of the value of evidence-informed decision-making, describing the latest research in bloggers’ area of expertise, and providing advice on strategies to improve the use of such research in decision-making; the blog style caters to the needs of policymakers by being engaging, current, relevant, practical and concise
Community	As required	Bulletin board where users can post information for other community members, such as new research, jobs or opportunities	User-driven	Provides a forum where policymakers and researchers can communicate and connect, access research, disseminate relevant research studies to policymakers, and discuss its relevance to current policy decisions
Research Tools	As required	Links to sources that provide high-level advice and methods on accessing research, appraising research and applying it to policy, and generating new research	Updated as required only; sources are those identified as high quality by CIPHER investigators and Web CIPHER users	Section provides links to key articles that provide methods on how to find research, and appraise the relevance and quality of research, as well as links to services offered by the Sax Institute for generating new research and reviews (when available evidence is lacking)
Research Portal	As required	Links to sources of high quality research and data, such as the Cochrane Library, the Campbell Collaboration, health-evidence.ca, Eppi-CENTRE, NIHR Centre for Reviews and Dissemination, the Australian Institute of Health and Welfare and the Australian Bureau of Statistics	Updated as required only; sources are those identified as high quality by CIPHER investigators and Web CIPHER users	Section provides links to key websites that provide one-stop shopping for systematic reviews, evidence briefs, high quality research journals, databases, and reliable sources of data and statistics; the section can be particularly helpful to staff whose organisations do not provide tools and systems that assist them in searching for and accessing research

Our preliminary research indicated that Web CIPHER showed great potential to improve how policymakers engaged with research [17]. Data revealed that over a 16-month period, 223 users from policymaking organisations joined the website. Six organisations accounted for more than 60% of the total number of users that joined. Five of these organisations were state agencies, each based in New South Wales. The other was a federal agency (now defunct) based in the Australian Capital Territory. Three of the six agencies focused on specific fields in health (e.g. cancer, drug prescribing), whereas the other three worked more broadly across public health and health systems improvement.

Viewing time and bounce rates (i.e. the percentage of users leaving the website after viewing a single page) on Web CIPHER were well above average for most websites [18]. The Blogs section was the most popular section on the site, followed by the Bulletin Board. Furthermore, a weekly email alerting members to newly posted Hot Topics, Research Updates and Blogs significantly increased the likelihood that users accessed Web CIPHER content, particularly those posts relating to new research.

We surmised that the Blogs were particularly popular for a number of key reasons. Firstly, they were written by trusted figures in health policy and research, which may have increased users’ perceived trust in the site, and their sense of community and social presence, all of which are predictive of website use [19–23]. Research has shown that staff are more likely to use knowledge platforms if they are perceived as credible, and are championed by trusted, recognised and authoritative figures [9, 24].

The blogs were also written in a clear and concise manner, with minimal jargon, and a conversational tone [14, 25], akin to the style of newspaper reports [26]. They were also delivered directly to users’ inbox via a weekly newsletter [27], and presented in a format suitable for mobile phones, tablets or related devices [26, 28]. The blogs often summarised relevant research findings [25, 29] and provided clear and concrete recommendations [14, 30]. Consequently, the blogs addressed some of the key barriers to research use such as limitations in policymakers’ research skills, complex presentation of research findings, excessive use of jargon and policy irrelevance [13, 14, 31].

In light of these findings, we aimed to test a number of strategies to further increase policymakers’ Web CIPHER usage, and hence their engagement with research evidence. Firstly, we aimed to examine the effect on usage of increasing both (1) the number of blogs posted on the website, and (2) the relevance of blogs by focusing on topics relating to particular agencies’ goals and missions, and electing trusted figures within those agencies to author some of the blogs. Secondly, we aimed to examine the impact of increasing the number of articles (i.e. hot topics and research updates) relevant to particular agencies’ missions, goals and purpose. Testing the impact of these interventions would provide valuable evidence on possible strategies to enhance organisations’ usage of knowledge platforms and improve policymakers’ engagement with research in their work.

Based on the evidence and mechanisms described above regarding factors that promote both website and research use, the present study had a number of hypotheses:

1. There will be a gradual increase in general Web CIPHER usage prior to and throughout the intervention period across all member organisations, as our previous research showed steady increases in the number of members throughout this period [17].
2. Increases in the number of blogs and tailored articles (i.e. hot topics and research updates) posted during the study period would each be associated with increases in general Web CIPHER usage across all policy agencies across the study period.
  - This effect, however, would be greater for blogs compared to articles.
3. Agencies will exhibit more sustained increases in general Web CIPHER usage following publication of blogs and articles targeted at their agency, relative to blogs and articles targeted at other agencies.
4. Agencies will exhibit sustained increases in general Web CIPHER usage following publication of blogs written by someone internal to their agency, compared to someone external to it.
5. In terms of usage for specific sections, the number of page views will be higher for blogs targeted at one’s agency, compared to blogs targeted at other agencies. This effect will be greater for blogs written by someone internal versus external to one’s agency.
6. The number of page views will be higher for articles targeted at one’s agency compared to articles targeted at other agencies.

## Methods

### Sample

Employees from numerous state and federal-level health agencies that expressed interested in joining the CIPHER network were invited by email to join Web CIPHER (see [17] for details). Because we were only interested in usage by ‘policymakers’, we created a custom segment of members from policy agencies only by excluding usage data from the web developers as well as members from universities and other research institutes (including the Sax Institute).

We selected the five agencies that had the highest number of members registered to the site prior to the study period, and who were not currently involved in a separate intervention trial called SPIRIT (Supporting Policy In health with Research: an Intervention Trial), which aims to increase policy agencies’ capacity to use research in policymaking [32]. The agencies were all Australian-based. Furthermore, each specialised in distinct areas of health, such as work health and safety, sexual health and cardiovascular health (Table 2), which enabled the posting of blogs specific to the goals and missions of that particular agency.

**Table 2:** Information regarding target agencies

Agency	Country based	Type of agency	Broad mission	Topic areas relevant to this agency
1	Australia	Independent, board-governed statutory authority	Undertaking activities to support the accountability of the state’s healthcare system	Reporting on performance of the health system, including safety and quality, effectiveness, efficiency and responsiveness of the system to people’s needs
2	Australia	Federal charity	Provision of information and support to enable Australians to look after their health	Cardiovascular health, cardiovascular disease (heart, stroke, blood vessel disease), treatment and prevention of cardiovascular disease
3	Australia	State and Federal government funded community health organisation	Providing access to quality information regarding reproductive and sexual health	Contraception, pregnancy options, sexually transmissible infections, sexuality and sexual function, menstruation, menopause, common gynaecological and vaginal problems, cervical screening, breast awareness, and men’s sexual health
4	Australia	State government agency	Protecting the health and safety of working Australians	Work health and safety in the workplace
5	Australia	State government agency	Provision of high quality clinical care and health-related transport services	Frontline delivery of clinical care (i.e. paramedics, counter disaster, special operations) and health-related transport services (including aeromedical and medical retrieval) to people in need

### Procedure

The intervention period for each target agency was 1 month, where 50% of the articles posted would focus on the topic area specific to that agency. The first agency was targeted during November–December 2014. The remaining agencies’ interventions were postponed until February 2015 due to the holiday period.

During an agency’s intervention month, two blogs were posted relating to the agency’s topic area: one by a staff member within the agency and the other by an external author. It was intended that blogs be posted 2 weeks apart within the agency’s month. Due to logistical reasons, however, blogs were posted outside of schedule, and only eight of the ten blogs were completed prior to analysis.

**Fig. 1:**
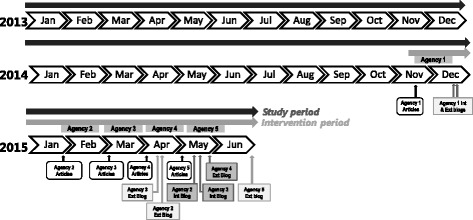
Study timeline illustrating the study and intervention periods, the months dedicated to each agency, and the publication point of each agency’s targeted articles and internally (Int.) and externally (Ext.) written blogs

The entire intervention took place over an 8-month period from November 2014 to June 2015. We labelled this period the Intervention Period. A detailed timeline of the intervention is displayed in Fig. 1, which indicates the points where agency-specific articles and blogs were posted.

### Data analysis

We tracked Web CIPHER usage from January 1, 2013, to June 30, 2015, using Google Analytics. We labelled this space in time the Study Period (Fig. 1). During this period we tracked daily usage of policy agencies using the number of new sessions as the outcome measure. Google Analytics defines a session as active interaction with the website (e.g. viewing different pages, posting comments) within a given time frame. A session ends when there is more than 30 minutes of inactivity, or if the user leaves the site and returns via a different route (e.g. an email link or search engine).

Time series analysis was used to examine members’ Web CIPHER usage throughout the Study Period. The auto-regressive, integrated, moving average or ARIMA (*p, d, q*)(*P, D, Q*)_*s*_ model (incorporating non-seasonal and seasonal trends) was used to model the change in usage over time and test whether the publication of articles and blogs on specific topics produced significant effects on usage [33]. We used the SPSS Expert Modeller to identify the ARIMA model for each time series analysis [34]. The modeller prints out values for the number of autoregressive (AR: *p*, *P*), moving average (MA: *q*, *Q*) and trend terms (*d, D*) and estimates any significant AR and MA parameters for both non-seasonal and seasonal components, as well as significant predictors of the outcome variable (i.e. usage).

To test hypotheses 1 and 2, we applied the expert modeller to the entire study period to firstly test whether usage of Web CIPHER increased over time, and secondly, whether this increase was associated with the number of tailored articles and blogs posted on the site during the Intervention Period. To address hypotheses 3 and 4, we conducted separate time-series analyses for each of the target agencies. Additional tests were conducted to determine if temporary and sustained increases in usage occurred following the publication of specific tailored article topics and blogs.

To test hypothesis 5, we conducted a three-way general loglinear analysis. General loglinear analysis is a statistical technique used to examine whether there is a relationship between multiple categorical variables [35]. In our analysis, there were three categorical variables: Agency of user (five levels: Agencies 1–5); Topic (two levels: targeted versus not-targeted to one’s agency); and Blog Author (two levels: Internal versus External to one’s agency). The number of page views was the dependent variable.

Prior to conducting analysis, it was observed that the number of page views for blogs and articles were generally quite low, with some blogs and articles not receiving any page views. The consequence of such low frequencies in a general loglinear analysis is a considerable reduction in statistical power, such that significant relationships between agency and agency-targeted blogs or articles would be harder to detect [36]. Importantly, however, low page view frequencies do not increase the Type I error rate (i.e. the likelihood of incorrectly concluding that there is a relationship when there is not). Therefore, we chose to accept reduced power for testing the relationship between agency and blog/article topic area.

To conduct the general loglinear analysis, we firstly fit a saturated model to test all main effects and interactions. The saturated model allowed us to test for significant relationships among all combinations of variables. This included the two-way relationships between Agency of user and Topic, Topic and Blog Author, Agency of user and Blog Author, the three-way relationship between Agency of user, Topic and Blog Author, and the effects of each variable alone (i.e. main effects). When no effects emerged as significant, we fit a hierarchical unsaturated model. An unsaturated model retains all variables in the analysis although certain combinations of variables are not tested. In our analysis, we removed the three-way interaction (Agency of user × Topic × Blog Author) and all two-way relationships involving Agency [35].

To test hypothesis 6, a χ^2^ analysis was conducted with two factors: Agency of user (five levels: Agencies 1–5) and Topic (two levels: targeted or not-targeted to the agency). This analysis allowed us to test whether agencies were more likely to view articles specific to their agency, and if this effect was only specific to particular agencies.

## Results

### Usage of Web CIPHER by all members

During the study period, 72% of Web CIPHER users came from policy agencies. A total of 392 policymakers from 97 organisations joined Web CIPHER during the study period. Policymakers from eight of the organisations accounted for more than 50% of the new users that joined during this period.

### Pre-intervention Web CIPHER usage by all members

**Fig. 2:**
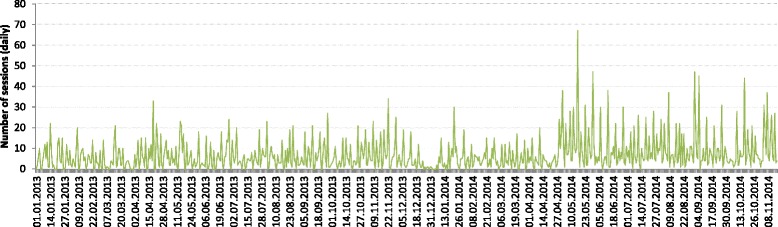
Baseline usage prior to the Intervention Period

To test hypothesis 1, a time-series model was estimated for the baseline (pre-intervention) period from January 1, 2013, to November 16, 2014. The length of this period was 685 days. As can be seen in Fig. 2, the variance of scores appears to be increasing over time. There were no obvious outliers. A log transformation was applied to the series. The expert modeller was applied to the baseline time series, which identified a seasonal ARIMA (0, 0, 4)(0, 1, 1)_7_, with differencing at lag 7 (i.e. every 7 days) required to achieve stationarity (Additional file 1). The model indicated a linear trend in the seasonal aspect of the model – that is, a gradual increase in the number of Web CIPHER sessions opened on the same day each week – each Friday when the weekly newsletter was sent out to users. There were three significant seasonal MA parameters at lags 1, 3 and 4 (Additional file 2).

### Impact of the number of articles and blogs on usage by all members

The ARIMA models for effects tested on all members are displayed in Additional file 1. Significant seasonal and non-seasonal AR and MA parameters for time series analyses examining usage by all members are provided in Additional file 2. Significant predictors in each of the subsequent analyses are displayed in Table 3.

**Table 3:** Significant moving average and autoregressive parameters and significant predictors (Articles and Blogs) for all members

Parameters/Predictors	Lag	Estimate	SE	t-statistic	sig
Impact of the number of articles and blogs on usage by all members					
Number of articles	0	0.080	0.026	3.090	0.002
	2	0.064	0.026	2.481	0.013
Impact of tailored articles on usage by all members – temporary effects					
Articles on Agency 3’s topic	0	0.854	0.263	3.245	0.001
Articles on Agency 5’s topic	1	0.671	0.265	2.536	0.011
Impact of tailored articles on usage by all members – sustained effects					
Articles on Agency 3’s topic	0	0.221	0.047	4.654	<0.001
Impact of tailored external blogs on usage by all members – temporary effects					
External blog on Agency 1’s topic	7	–0.668	0.261	−2.561	0.011
External blog on Agency 2’s topic	1	0.826	0.263	3.143	0.002
External blog on Agency 4’s topic	9	0.666	0.264	2.519	0.012
Impact of tailored internal blogs on usage by all members – temporary effects					
Internal blog on Agency 1‘s topic	0	–0.665	0.261	−2.545	0.011
Internal blog on Agency 2’s topic	0	0.650	0.263	2.468	0.014
Internal blog on Agency 3’s topic	0	0.546	0.264	2.066	0.039

**Fig. 3:**
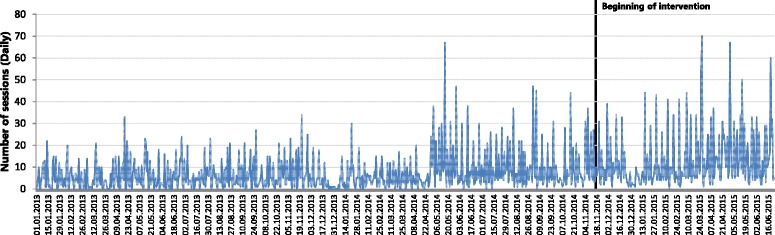
Daily number of Web CIPHER sessions across the Study Period and Intervention Period for users across all member organisations

The daily number of Web CIPHER sessions across the entire study period (including the intervention period) across all member organisations is displayed in Fig. 3. To test hypothesis 2, the expert modeller was applied to usage over the entire study period and generated a seasonal ARIMA model (0, 0, 3)(0, 1, 1)_7_. Differencing was required at lag 7 to achieve stationarity, indicating a linear trend in usage over time. Contrary to our hypothesis, the number of articles, but not blogs, emerged as a significant positive predictor of usage over time (Table 3). The effect of articles was significant at lags 0 and 2 and had a delay of 7 days. This meant that, at any point in time, the number of articles currently on the site and 2 weeks earlier, were significantly predictive of greater usage 7 days later.

### Impact of the intervention on usage by all members

The results above showed that Web CIPHER usage across all member organisations was generally increasing over time, and this was uniquely associated with increases in the number of articles (but not blogs) posted over time. We now turn to examining the effect of the intervention on all members’ usage.

#### Impact of tailored articles on usage by all members

The expert modeller generated a seasonal ARIMA model (0, 0, 3)(1, 1, 1)_7_. Interestingly, the results showed that the date at which the first Agency 3 and Agency 5 topics were published led to a significant, temporary increase in usage (Table 3). Anti-logging the intervention parameters (i.e. *ω*), indicated that the point at which Agency 3 articles were published increased the number of sessions by 6.145, whereas the point at which Agency 5 articles were first published increased the number of sessions by 3.688 sessions.

We next tested whether any of these observed impacts were sustained, following the coding guidelines of Tabachnick and Fidell [36]. The ARIMA model was re-estimated and a significant ARIMA model of (0, 0, 3)(0, 1, 1)_7_ emerged. Agency 3 topic articles produced a highly significant and sustained effect on Web CIPHER usage (Table 3). Anti-logging the intervention parameter revealed that there was sustained increase of 0.663 Web CIPHER sessions per day for the remainder of the study period, following the publication of articles targeting Agency 3.

#### Impact of tailored blogs on usage by all members

We first looked at the impact of the externally written blogs on the number of Web CIPHER sessions for all members. The ARIMA model was re-estimated and a significant ARIMA model of (0, 0, 3)(0, 1, 1)_7_ emerged. Results revealed that the Agency 1, Agency 2 and Agency 4 blogs were followed by significant, temporary shifts in member organisations’ usage (Table 3). Anti-logging the intervention parameters, showed that usage dropped by 0.79 sessions when the Agency 1 blog was published. However, usage increased temporarily by 5.599 sessions and by 3.634 sessions when the blogs for Agency 2 and 4, respectively, were published. Further analyses revealed, however, that none of these effects were sustained (Table 3).

We repeated the above analysis, this time testing the impact of the internally written blogs on Web CIPHER sessions. The ARIMA model was re-estimated and an ARIMA model of (0, 0, 3)(0, 1, 1)_7_ emerged. Results showed that the Agency 1, Agency 2 and Agency 4 blogs were followed by significant, temporary shifts in usage across all member organisations (Table 3). Anti-logging the intervention parameters revealed that usage dropped by 0.79 sessions when the Agency 1 internal blog was published. However, usage temporarily increased by 3.467 sessions and by 2.516 sessions when the internal blogs for Agencies 2 and 3, respectively, were published. Additional analyses revealed, however, that these effects were not sustained (Table 3).

### Impact of the intervention on usage by the target agencies

We next examined the impact of the intervention on the usage of each of the five target agencies (i.e. Agencies 1 to 5). The outcome variable was the number of Web CIPHER sessions opened throughout the study period for a particular agency. The aim was to test hypothesis 3, that agencies’ usage of Web CIPHER would be more likely to increase following the publication of articles and blogs that are targeted, versus not targeted, at one’s own agency. ARIMA models generated for each targeted agency for all subsequent analyses are displayed in Additional file 3, and significant seasonal and non-seasonal AR and MA parameters are provided in Additional file 4. Significant predictors in each of the subsequent analyses are displayed in Table 4.

**Table 4:** Significant moving average and autoregressive parameters and significant predictors (Articles and Blogs) for users across in the target agencies

Agency targeted	Predictor	Lag	Estimate	SE	*t*-statistic	sig
Temporary impact of agency-specific articles on usage						
Agency 3	Articles on Agency 5’s topic	0	0.867	0.320	2.712	0.007
Sustained impact of agency-specific articles on usage						
Agency 3	Articles on Agency 3’s topic	0	0.189	0.056	3.380	0.001
	Articles on Agency 4’s topic	0	–0.183	0.064	−2.853	0.004
Temporary impact of external authored agency-specific blogs on usage						
Agency 5	Blogs on Agency 4’s topic	0	0.724	0.302	2.396	0.017
Temporary impact of internal authored agency-specific blogs on usage						
Agency 5	Blogs on Agency 2’s topic	0	0.730	0.300	2.434	0.015

#### Impact of tailored articles on targeted agencies’ usage

Examination of the time-series plots for each agency indicated that the variance of scores was relatively stable over the study period and so data transformation was not necessary prior to analysis. As shown in Table 4, Agency 3 showed a significant temporary increase in usage when articles targeting Agency 5 were posted, by 0.867 sessions. Agency 2 showed a significant temporary increase in usage when articles targeting Agency 4 were posted, by approximately 1 session.

We next examined whether agencies showed sustained increases in their Web CIPHER usage. As shown in Table 4, Agency 3 showed a significant and sustained increase in usage following articles published relating to their own topic by 0.189 sessions per day. Conversely, they showed a significant drop in usage by 0.183 sessions following articles relating to Agency 4.

#### Impact of the blogs on targeted agencies’ usage

##### Externally authored blogs

Time series models were run for each of the five agencies with the publication date of the four externally authored blogs entered as predictors. As Shown in Table 4, Agency 5 showed a significant temporary increase in usage by 0.987 sessions, following the blog on Agency 4’s topic. There were no sustained effects of the external blogs on any of the five target agencies’ site usage.

##### Internal blogs

Time series models were run for each of the five target agencies with the publication date of the three internally authored blogs entered as predictors. As shown in Table 4, only Agency 4 showed a significant abrupt increase in usage following the blog written in relation to Agency 2, by 0.730 sessions. Contrary to hypothesis 4, the analyses showed that there were no sustained effects of the internal blogs on any of the five target agencies.

### Relationship between agency, topic area and blog author on the number of page views for blogs among existing users

We conducted general loglinear analyses to examine whether page views would be higher for blogs targeted at one’s agency, compared to blogs targeted at other agencies, and whether this effect would be greater if these blogs were written by someone internal, versus external to one’s agency (hypothesis 5). The results revealed two significant effects of Agency – that is, views of blogs were significantly higher for Agency 1, *z* = 2.391, *P* = 0.017, and Agency 3, *z* = 2.868, *P* = 0.004, relative to the reference group (Agency 5). Table 5 shows that Agency 1 (33.9% of total views) and Agency 3 (40.7% of total views) had the highest overall blog views compared to the remaining agencies. There was no effect of blog topic, meaning that views did not differ between blogs targeted versus not targeted at one’s agency. There was, however, a significant effect of author, where users were more likely to view blogs written by someone internal versus external to their agency. Of greatest interest was the significant interaction between blog topic and author, *z* = 3.145, *P* = 0.002. A two-way cross tabulation of this effect shown in Table 6 revealed that page views were higher for targeted versus non-targeted blogs, and that this effect was significantly greater when the blog author was internal versus external to the agency.

**Table 5:** Number of page views for internally authored and externally authored blogs for users in each of the target agencies

		Blog targeted at agency	Blog targeted at other agencies	
		Page views (% of total)	Page views (% of total)	Total
Agency 1	Internal author	8 (13.6)	0 (0)	8 (13.6)
	External author	12 (20.3)	0 (0)	12 (20.3)
Agency 2	Internal author	2 (3.4)	0 (0)	2 (3.4)
	External author	0 (0)	0 (0)	0 (0)
Agency 3	Internal author	20 (33.9)	2 (3.4)	22 (37.3)
	External author	0 (0)	2 (3.4)	2 (3.4)
Agency 4	Internal author	0 (0)	2 (3.4)	2 (3.4)
	External author	0 (0)	4 (6.8)	4 (6.8)
Agency 5	Internal author	0 (0)	0 (0)	0 (0)
	External author	0 (0)	7 (11.9)	7 (11.9)
Total		42 (72)	17 (29)	59 (100)

**Table 6:** Cross-tabulation between the topic specificity and blog author on the number of page views

		Author		Total, n (%)
		Internal, n (%)	External, n (%)	
Topic	Agency targeted	30 (50.8)	12 (20.3)	42 (71.2)
	Non-Agency targeted	4 (6.8)	13 (22.0)	17 (28.8)
Total		34 (57.6)	25 (42.4)	59 (100)

### Relationship between agency and topic area on number of page views for articles among existing users

We next tested hypothesis 6, that page views among existing users from target agencies would be higher for targeted versus non-targeted articles. The number of page views of agency-targeted versus non-targeted articles for each of the five agencies is displayed in Table 7. The two-way general loglinear analysis revealed a significant effect for Agency 3, *z* = −58.794, *P* < 0.001. From the row totals, we can see that 68% of article views were by users from Agency 3. Of greatest importance was the significant effect of topic, *z* = 1.674, *P* = 0.008, such that there were a higher number of views for articles targeted at one’s agency compared to articles targeted at other agencies. The column totals showed that 91.7% of total article views were for articles targeted at one’s agency. There was no significant interaction between agency and topic.

**Table 7:** Number of page views for targeted versus non-targeted blogs for users in each of the target agencies

	Topic		Total, n (%)
	Agency targeted, n (%)	Non-agency targeted, n (%)	
Agency 1	0 (0.0)	1 (0.9)	1 (0.9)
Agency 2	0 (0.0)	1 (0.9)	1 (0.9)
Agency 3	74 (67.9)	0 (0.0)	74 (67.9)
Agency 4	10 (9.2)	4 (3.7)	14 (12.8)
Agency 5	16 (14.7)	3 (2.8)	19 (17.4)
Total	100 (91.7)	9 (8.3)	109 (100.0)

## Discussion

The present paper documents the findings of an innovative study examining the impact of agency-tailored articles and blogs on policymakers’ usage of Web CIPHER, an online portal that aims to increase policymakers’ engagement and interest in research. The results provide us with insights into strategies to improve policymakers’ engagement with research using such online platforms but indicate that there is much still to be learnt.

We found mixed support for our hypotheses. In support of hypothesis 1, there was an increase in Web CIPHER usage across all member organisations prior to the intervention. Contrary to hypothesis 2, Web CIPHER usage among member organisations was significantly associated with the number of new articles posted but not the number of blogs posted across the intervention period. Articles on topics relevant to Agency 3 (relating to sexual and reproductive health) were the only articles to produce a sustained increase in usage across all agencies. There was slight support for hypothesis 3, as only Agency 3 showed sustained increases in usage following articles (and not blogs) specifically targeted at their own agency, and not following articles and blogs targeted at other agencies. There was no support for hypothesis 4, as neither the internally- nor externally-authored blogs led to sustained increases in usage for any agency. Three target agencies (2, 4 and 5) showed significant increases in usage following blogs and articles directed at other agencies although these increases were only temporary. In support of hypotheses 5 and 6, loglinear analyses revealed that, among the existing Web CIPHER users from the target agencies, page views for articles and blogs targeted at one’s agency were higher than those targeted at other agencies. This effect was stronger for internally- versus externally-authored blogs. This indicates that, although blogs did not increase Web CIPHER usage in general, users were more likely to view blogs targeted at their agency compared to other agencies, particularly when these were written by someone internal to their agency.

The first key finding was that usage among all member organisations increased as more articles (hot topics and research updates) were posted throughout the intervention period. It stands to reason that the more content that is posted the greater the likelihood of content being of interest to users, thereby prompting members to access the website. This suggests that usage may have been motivated by hedonic goals (i.e. a desire to experience fun, pleasure and enjoyment [37]), which has been shown to be a significant driver of site usage. This is supported by the fact that the hot topics in particular were about new developments and initiatives that users likely did not know already, which would have contributed to their appeal. Because Web CIPHER is not set up to be a one-stop shop for research, it is unlikely that users were driven by utilitarian motivations such as the perceived usefulness of the information for achieving one’s work-related goals.

Further support for the hedonic explanation comes from the fact that there was a sustained increased in usage across all member organisations following articles posted about sexual and reproductive health (i.e. Agency 3’s topic). It is likely that users found these topics interesting to read about and personally relevant even though they were not necessarily relevant to most members’ work. It is well-documented that ‘sex sells’ [38]; therefore, it is likely that Agency 3 articles were regarded as both appealing and personally relevant to users across all member organisations, which helped drive sustained increases in Web CIPHER usage.

Contrary to what was predicted, the number of blogs posted was not uniquely predictive of greater Web CIPHER usage. On the surface, these results are surprising in light of our previous study [17], which found that blogs were the most popular section on the website. However, the blogs analysed in our original study were not specifically tailored to individual agencies. Rather, they were designed to be of general interest, focusing on broad topics such as the value of research in policy and skills to facilitate dissemination and translation [17]. In contrast, in the present study, the blogs were tailored to specific agencies and fewer in number, and so were less likely to have broad appeal to users. Although the articles were also tailored to agencies, there was a large number posted throughout the intervention period (almost 100), meaning that there was likely to be something that sparked users’ interest and promoted greater usage. These findings lead us to infer that blogs in and of themselves might not necessarily be compelling enough to promote greater usage of such knowledge portals. Rather, for content to promote usage, it needs to be widely appealing, thought-provoking, interesting and, ideally, abundant and frequently updated. Further research is required to verify this conclusion.

Content relevance also influenced site usage to some degree. Firstly, the loglinear analyses showed that articles and blogs that were targeted to one’s agency had significantly greater page views than those targeted at other agencies. Secondly, Agency 3 exhibited a sustained increase in usage following the articles targeted to their agency. Such specific and sustained effects were not observed in other agencies, however. This indicates that agencies were more likely to read articles (and internal blogs) relevant to their agency, although these targeted articles only produced sustained increases in general Web CIPHER for Agency 3. It is possible that the articles posted in this period were directly relevant to the work and projects of Agency 3 staff at the time, thus encouraging them to continue visiting the site over the subsequent weeks. When articles on Agency 4 were posted, Agency 3’s usage significantly declined. These conclusions are tentative in light of the fact that similar effects did not emerge in any of the other agencies, demonstrating that further work is required to tease out the specific conditions under which relevance impacts upon usage of knowledge platforms.

Our results also provided some evidence for the impact of familiar authors on usage behaviour of agencies. Specifically, it was found that Agency 3 users showed a significant increase in usage following a blog written by an author internal but not external to the agency. This effect was temporary, however, and did not occur for the remaining target agencies. We predicted this effect would emerge across all agencies, because the internal authors would be more familiar, trusted and credible [19, 21, 23], focus on agency-relevant topics, represent more obvious champions of research use for their agencies [9], and trigger a greater sense of social presence and critical mass, relative to authors external to the agency. Something perceived as familiar might, on the other hand, be interpreted as mundane and repetitive. A familiar individual might also not necessarily be supported or positively regarded by users. Until further research is undertaken, firm conclusions regarding the impact of author familiarity on usage of such portals cannot be made.

Many organisations are now adopting the use of online knowledge platforms to help their staff better access, store, retrieve and exchange information across the organisation, and thereby improve organisational performance and adherence to best practices [24, 39, 40]. Knowledge platforms could therefore be used to improve policymakers’ access, retrieval and exchange of research, thereby encouraging greater engagement with and use of research in their work [17]. The present findings have implications for strategies to increase policymakers’ engagement with research through such knowledge platforms. Given that our study is the first of its kind, many of these findings are tentative, but nonetheless consistent with the extant research on factors that promote usage of websites and knowledge platforms [8, 24, 39, 40]. Firstly, our findings suggest that there needs to be an abundance of widely relevant and regularly updated content on a website to provoke use. Our findings indicate that this content must be relevant (personally or professionally), but must also be interesting (i.e. novel, enjoyable, compelling, engaging, thought-provoking, appealing or stimulating) [22, 23, 41–44]. Therefore, in order to attract policymakers and increase their engagement with health research, the content has to stimulate, entertain and engage akin to most forms of mass media, as well as be relevant. There should ideally be an abundance of content and it may also help if this content is written about or by experts in policy and health who are widely regarded and trusted.

Our findings further suggest that blogs in and of themselves are not uniquely predictive of greater use. We propose that, for blogs to promote usage, they must also be widely appealing, engaging and interesting (as suggested by our previous research [17]), rather than being highly specific and targeted to a particular agencies’ program of work. Given that the number of articles uniquely predicted greater usage over time, and that frequent blogs (e.g. once a week) would require a great deal of work to coordinate and produce, it is possible that blogs might not be worth the investment as a strategy to enhance usage of similar knowledge platforms. Further research is needed to tease this relationship out.

Our study represents the first attempt to undertake an in-depth, longitudinal investigation of policymakers’ usage of an online portal directed at health policymakers using time series analysis and Google Analytics. Ours is also the first to test the impact of a range of strategies to increase policymakers’ usage of such portals over time. Previous studies on the use of knowledge platforms and websites have primarily been cross-sectional in design and present correlational findings. In these studies, it is difficult to make definitive conclusions as to what strategies have causal impacts on staff engagement with such platforms. Interrupted time series analysis, however, allows one to examine behaviour (i.e. Web CIPHER usage) before and after an identifiable event or intervention (i.e. the posting of agency-tailored blogs and articles), in order to evaluate the impact of this behaviour [36, 45]. Although all threats to validity of causal inferences cannot be ruled out using such an analysis strategy [33], our study goes some way towards providing stronger conclusions about what strategies can be used to improve policymakers’ engagement with and use of such platforms.

The present study also had some limitations. Firstly, although there were a relatively large number of policymakers from all member organisations collectively (n = 392), there was a small number of policymakers from the individual target agencies. Selection bias may also have been an issue in the present study given that all users voluntarily elected to login and use the website, and so may have had pre-existing interests in knowledge platforms. Our conclusions may therefore only apply to this self-selected sample. Furthermore, the use of sessions as the main indicator of usage has issues, because it does not take into account the number of users opening these sessions. Specifically, it is possible that a single user could have opened all the recorded sessions, making sessions a somewhat biased indicator of usage. Also we had no control over historical events that occurred throughout the intervention period that may have influenced usage, aside from the intervention itself. For example, the blogs targeting Agency 1 were posted towards the end of 2014, prior to the Christmas holiday break. Agency 1 showed significant decreases in usage following the onset of these blogs, but this was most likely the result of the subsequent holiday period than the blogs themselves. Blogs were posted outside of their scheduled date due to a number of logistical reasons which may have reduced their potential impact on Web CIPHER usage among the target agencies.

## Conclusion

Knowledge platforms are a recent innovation that may potentially improve policymakers’ capacity to access and apply research to their work. It is important to mention, however, that knowledge platforms require agencies to have ready access to the appropriate technology such as computers, tablets, smartphones and high-speed internet access, which may not be the case for certain low- and middle-income countries [46]. Therefore, the findings presented here may only apply to health policy agencies in some countries. Nonetheless, we provide an innovative and novel investigation of the impact of strategies to increase policymakers’ usage of one such knowledge platform, known as Web CIPHER. Specifically, we examined the effect of publishing hot topics, research updates and blogs on topics of direct relevance to some of Web CIPHER’s member agencies. The results were highly variable, although a number of interesting trends emerged. Firstly, general usage of Web CIPHER gradually increased over time, and this was significantly predicted by the number of tailored articles (i.e. hot topics and research) that were posted throughout the Study Period, but not the number of blogs. Articles focused on sexual and reproductive health (but not other topics) were followed by sustained increases in usage for users across all member organisations. Further, only one of the five target agencies exhibited sustained increases in usage when articles were published directly targeting that agency. Finally, page views were higher for articles targeted at one’s agency versus other agencies. For blogs, this effect was stronger when the author was internal versus external to one’s agency. The findings suggest that usage was more strongly driven by interest, enjoyment, appeal and personal relevance, as opposed to agency specificity and work relevance.

In light of these mixed results, a number of tentative implications emerged regarding strategies to improve policymakers’ use of knowledge platforms. First and foremost, the content needs to be personally relevant, widely interesting, engaging, appealing to users and frequently updated, regardless of whether this content consists of news articles, research updates or blogs. Recruiting familiar and trusted authors could possibly augment the effectiveness of these strategies. Although further research is required, the present study goes some way towards better understanding what strategies might be effective for increasing policymakers’ engagement with research through the use of online knowledge platforms.

## Additional files

Additional file 1: ARIMA models for users from all member organisations. (DOCX 12 kb)
Additional file 2: Significant moving average and autoregressive parameters for all members. (DOCX 14 kb)
Additional file 3: ARIMA models for users from targeted agencies. (DOCX 13 kb)
Additional file 4: Significant moving average and autoregressive parameters for users in target agencies. (DOCX 16 kb)

## Abbreviations

AR: Autoregressive
ARIMA: Auto-regressive, integrated, moving average
CIPHER: Centre for Informing Policy in Health with Evidence from Research
MA: Moving average
