# Supplementary material for: Impact of tailored blogs and content on usage of Web CIPHER – an online platform to help policymakers better engage with evidence from research
Source: Health Res Policy Syst. 2016 Dec 1;14:85. doi: 10.1186/s12961-016-0157-5 (PMC5134066; doi:10.1186/s12961-016-0157-5)
Supplement: Additional file 1: — ARIMA models for users from all member organisations. (DOCX 12 kb) [file 12961_2016_157_MOESM1_ESM.docx]

Additional File 1
*ARIMA Models for Users from all Member Organisations*

| Model tested | ARIMA MODEL | Model Fit | | | |
| --- | --- | --- | --- | --- | --- |
|  |  | Stationary R-square | Ljung-Box Q Statistic | df | sig |
| Pre-intervention (baseline) | ARIMA(0,0,3)(0,1,1)_7_ | .424 | 15.362 | 14 | .354 |
| Impact of the number of articles and blogs | ARIMA(0,0,3)(0,1,1)_7_ | .470 | 25.381 | 15 | .045 |
| Temporary impact of specific article topics on usage | ARIMA(0,0,3)(1,1,1)_7_ | .455 | 35.073 | 14 | .001 |
| Sustained Impact of specific article topics on usage | ARIMA(0,0,3)(0,1,1)_7_ | .463 | 24.611 | 15 | .055 |
| Temporary impact of specific external blog topics on usage | ARIMA(0,0,3)(0,1,1)_7_ | .475 | 20.810 | 15 | .143 |
| Sustained of specific external blog topics on usage | ARIMA(0,0,3)(1,1,1)_7_ | .443 | 32.163 | 14 | .004 |
| Temporary impact of specific internal blog topics on usage | ARIMA(0,0,3)(0,1,1)_7_ | .472 | 25.131 | 15 | .048 |
| Sustained impact of specific internal blog topics on usage (sustained effect) | ARIMA(0,0,3)(1,1,1)_7_ | .443 | 32.163 | 14 | .004 |
