# Supplementary material for: Impact of tailored blogs and content on usage of Web CIPHER – an online platform to help policymakers better engage with evidence from research
Source: Health Res Policy Syst. 2016 Dec 1;14:85. doi: 10.1186/s12961-016-0157-5 (PMC5134066; doi:10.1186/s12961-016-0157-5)
Supplement: Additional file 2: — Significant moving average and autoregressive parameters for all members. (DOCX 14 kb) [file 12961_2016_157_MOESM2_ESM.docx]

Additional File 2
*Significant Moving Average and Autoregressive Parameters for all Members*

| Parameters/Predictors | Lag | Estimate | SE | t-statistic | sig |
| --- | --- | --- | --- | --- | --- |
| A. Pre-intervention Web CIPHER usage by all members | | | | | |
| MA | 1 | -.149 | .038 | -3.927 | <.001 |
|  | 3 | -.126 | .038 | -3.315 | .001 |
|  | 4 | -.145 | .038 | -3.773 | <.001 |
| MA, Seasonal | 1 | .873 | .021 | 42.417 | <.001 |
| B. Impact of the number of articles and blogs on usage by all members | | | | | |
| MA | 1 | -.136 | .033 | -4.121 | <.001 |
|  | 3 | -.122 | .033 | -3.682 | <.001 |
| MA, seasonal | 1 | .934 | .015 | 64.308 | <.001 |
| C. Impact of tailored articles on usage by all members – temporary effects | | | | | |
| MA | 1 | -.155 | .033 | -4.717 | <.001 |
|  | 3 | -.133 | .033 | -3.997 | <.001 |
| AR | 1 | .095 | .036 | 2.612 | .009 |
| MA, seasonal | 1 | .945 | .015 | 65.071 | <.001 |
| D. Impact of tailored articles on usage by all members – sustained effects | | | | | |
| MA | 1 | -.134 | .033 | -4.090 | <.001 |
|  | 3 | -.124 | .033 | -3.782 | <.001 |
| MA, seasonal | 1 | .941 | .014 | 67.846 | <.001 |
| E. Impact of tailored external blogs on usage by all members – temporary effects | | | | | |
| MA | 1 | -.136 | .033 | -4.113 | <.001 |
|  | 3 | -.119 | .033 | -3.588 | <.001 |
| MA, seasonal | 1 | .943 | .014 | 68.899 | <.001 |
| F. Impact of tailored external blogs on usage by all members – sustained effects | | | | | |
| MA | 1 | -.162 | .033 | -4.943 | <.001 |
|  | 3 | -.122 | .033 | -3.712 | <.001 |
| AR, seasonal | 1 | .099 | .036 | 2.719 | .007 |
| MA, seasonal | 1 | .946 | .014 | 66.160 | <.001 |
| G. Impact of tailored internal blogs on usage by all members – temporary effects | | | | | |
| MA | 1 | -.138 | .033 | -4.177 | <.001 |
|  | 3 | -.132 | .033 | -4.009 | <.001 |
| MA, seasonal | 1 | .942 | .014 | 67.738 | <.001 |
| H. Impact of tailored internal blogs on usage by all members – sustained effects | | | | | |
| MA | 1 | -.162 | .033 | -4.943 | <.001 |
|  | 3 | -.122 | .033 | -3.712 | <.001 |
| AR, seasonal | 1 | .099 | .036 | 2.719 | .007 |
