# Supplementary material for: Impact of tailored blogs and content on usage of Web CIPHER – an online platform to help policymakers better engage with evidence from research
Source: Health Res Policy Syst. 2016 Dec 1;14:85. doi: 10.1186/s12961-016-0157-5 (PMC5134066; doi:10.1186/s12961-016-0157-5)
Supplement: Additional file 3: — ARIMA models for users from targeted agencies. (DOCX 13 kb) [file 12961_2016_157_MOESM3_ESM.docx]

Additional File 3
*ARIMA Models for Users from Targeted Agencies*

| Model tested | ARIMA MODEL | Model Fit | | | |
| --- | --- | --- | --- | --- | --- |
|  |  | Stationary *R-*square | Ljung-Box Q Statistic | df | sig |
| Temporary impact of agency-specific articles on usage | | | | | |
| Agency 1 | ARIMA(0,0,0)(1,0,1)_7_ | .040 | 13.097 | 16 | .666 |
| Agency 2 | ARIMA(0,0,0)(1,0,1)_7_ | .458 | 12.226 | 17 | .786 |
| Agency 3 | ARIMA(1,0,1)(1,0,1)_7_ | .065 | 11.860 | 16 | .754 |
| Agency 4 | ARIMA(0,0,0)(0,1,1)_7_ | .153 | 18.833 | 14 | .171 |
| Agency 5 | ARIMA(0,0,0)(1,0,1)_7_ | .245 | 20.891 | 16 | .183 |
| Sustained impact of agency-specific articles on usage | | | | | |
| Agency 1 | ARIMA(0,0,0)(1,0,1)_7_ | .040 | 13.097 | 16 | .666 |
| Agency 2 | ARIMA(0,0,0)(1,0,1)_7_ | .449 | 11.712 | 17 | .817 |
| Agency 3 | ARIMA(1,0,1)(1,0,1)_7_ | .067 | 10.563 | 16 | .836 |
| Agency 4 | ARIMA(0,0,0)(0,1,1)_7_ | .153 | 18.833 | 14 | .171 |
| Agency 5 | ARIMA(0,0,0)(1,0,1)_7_ | .245 | 20.891 | 16 | .183 |
| Temporary impact of external authored agency-specific blogs on usage | | | | | |
| Agency 1 | ARIMA(0,0,0)(1,0,1)_7_ | .040 | 13.097 | 16 | .666 |
| Agency 2 | ARIMA(0,0,0)(1,0,1)_7_ | .449 | 11.712 | 17 | .817 |
| Agency 3 | ARIMA(1,0,1)(1,0,1)_7_ | .057 | 11.121 | 16 | .802 |
| Agency 4 | ARIMA(0,0,0)(0,1,1)_7_ | .153 | 18.833 | 14 | .171 |
| Agency 5 | ARIMA(0,0,0)(1,0,1)_7_ | .250 | 21.646 | 16 | .155 |
| Sustained impact of external authored agency-specific blogs on usage | | | | | |
| Agency 1 | ARIMA(0,0,0)(1,0,1)_7_ | .040 | 13.097 | 16 | .666 |
| Agency 2 | ARIMA(0,0,0)(1,0,1)_7_ | .449 | 11.712 | 17 | .817 |
| Agency 3 | ARIMA(1,0,1)(1,0,1)_7_ | .057 | 11.121 | 16 | .802 |
| Agency 4 | ARIMA(0,0,0)(0,1,1)_7_ | .153 | 18.833 | 14 | .171 |
| Agency 5 | ARIMA(0,0,0)(1,0,1)_7_ | .245 | 20.891 | 16 | .183 |
| Temporary impact of internal authored agency-specific blogs on usage | | | | | |
| Agency 1 | ARIMA(0,0,0)(1,0,1)_7_ | .040 | 13.097 | 16 | .666 |
| Agency 2 | ARIMA(0,0,0)(1,0,1)_7_ | .449 | 11.712 | 17 | .817 |
| Agency 3 | ARIMA(1,0,1)(1,0,1)_7_ | .057 | 11.121 | 16 | .802 |
| Agency 4 | ARIMA(0,0,0)(0,1,1)_7_ | .153 | 18.833 | 14 | .171 |
| Agency 5 | ARIMA(0,0,0)(1,0,1)_7_ | .250 | 19.743 | 16 | .232 |
| Sustained impact of internal authored agency-specific blogs on usage | | | | | |
| Agency 1 | ARIMA(0,0,0)(1,0,1)_7_ | .040 | 13.097 | 16 | .666 |
| Agency 2 | ARIMA(0,0,0)(1,0,1)_7_ | .449 | 11.712 | 17 | .817 |
| Agency 3 | ARIMA(1,0,1)(1,0,1)_7_ | .057 | 11.121 | 16 | .802 |
| Agency 4 | ARIMA(0,0,0)(0,1,1)_7_ | .153 | 18.833 | 14 | .171 |
| Agency 5 | ARIMA(0,0,0)(1,0,1)_7_ | .245 | 20.891 | 16 | .183 |
