# Supplementary material for: Impact of tailored blogs and content on usage of Web CIPHER – an online platform to help policymakers better engage with evidence from research
Source: Health Res Policy Syst. 2016 Dec 1;14:85. doi: 10.1186/s12961-016-0157-5 (PMC5134066; doi:10.1186/s12961-016-0157-5)
Supplement: Additional file 4: — Significant moving average and autoregressive parameters for users in target agencies. (DOCX 16 kb) [file 12961_2016_157_MOESM4_ESM.docx]

Additional File 4
*Significant Moving Average and Autoregressive Parameters for Users in Target Agencies*

| Parameters/Predictors | | Lag | Estimate | SE | t-statistic | sig |
| --- | --- | --- | --- | --- | --- | --- |
| A. Temporary impact of agency-specific articles on usage | | | | | | |
| Agency 1 | | | | | | |
|  | AR, seasonal | 1 | .992 | .004 | 264.098 | <.001 |
|  | MA, seasonal | 1 | .934 | .015 | 60.943 | <.001 |
| Agency 2 | |  |  |  |  |  |
|  | MA, seasonal | 1 | .864 | .018 | 48.773 | <.001 |
| Agency 3 | | | | | | |
|  | AR, seasonal | 1 | 1.000 | .002 | 509.310 | <.001 |
|  | MA, seasonal | 1 | .993 | .020 | 50.409 | <.001 |
| Agency 4 | | | | | | |
|  | AR | 1 | .917 | .048 | 18.987 | <.001 |
|  | MA | 1 | .857 | .062 | 13.815 | <.001 |
|  | AR, seasonal | 1 | .997 | .002 | 492.591 | <.001 |
|  | MA, seasonal | 1 | .965 | .012 | 77.726 | <.001 |
| Agency 5 | | | | | | |
|  | AR, seasonal | 1 | .987 | .006 | 167.695 | <.001 |
|  | MA, seasonal | 1 | .896 | .019 | 47.582 | <.001 |
| B. Sustained impact of agency-specific articles on usage | | | | | | |
| Agency 1 | | | | | | |
|  | AR, seasonal | 1 | .992 | .004 | 264.098 | <.001 |
|  | MA, seasonal | 1 | .934 | .015 | 60.943 | <.001 |
| Agency 2 | | | | | | |
|  | MA, seasonal | 1 | .866 | .018 | 49.358 | <.001 |
| Agency 3 | | | | | | |
|  | AR, seasonal | 1 | 1.000 | .001 | 669.897 | <.001 |
|  | MA, seasonal | 1 | .991 | .019 | 53.281 | <.001 |
| Agency 4 | | | | | | |
|  | AR | 1 | .917 | .048 | 18.987 | <.001 |
|  | MA | 1 | .857 | .062 | 13.815 | <.001 |
|  | AR, seasonal | 1 | .997 | .002 | 492.591 | <.001 |
|  | MA, seasonal | 1 | .965 | .012 | 77.726 | <.001 |
| Agency 5 | | | | | | |
|  | AR, seasonal | 1 | .987 | .006 | 167.695 | <.001 |
|  | MA, seasonal | 1 | .896 | .019 | 47.582 | <.001 |
| C. Temporary impact of external authored agency-specific blogs on usage | | | | | | |
| Agency 1 | | | | | | |
|  | AR, seasonal | 1 | .992 | .004 | 264.098 | <.001 |
|  | MA, seasonal | 1 | .934 | .015 | 60.943 | <.001 |
| Agency 2 | | | | | | |
|  | MA, seasonal | 1 | .866 | .018 | 49.358 | <.001 |
| Agency 3 | | | | | | |
|  | AR, seasonal | 1 | .999 | .003 | 377.057 | <.001 |
|  | MA, seasonal | 1 | .988 | .018 | 55.583 | <.001 |
| Agency 4 | | | | | | |
|  | AR | 1 | .917 | .048 | 18.987 | <.001 |
|  | MA | 1 | .857 | .062 | 13.815 | <.001 |
|  | AR, seasonal | 1 | .997 | .002 | 492.591 | <.001 |
|  | MA, seasonal | 1 | .965 | .012 | 77.726 | <.001 |
| Agency 5 | | | | | | |
|  | AR, seasonal | 1 | .987 | .006 | 166.109 | <.001 |
|  | MA, seasonal | 1 | .896 | .019 | 47.472 | <.001 |
| D. Sustained impact of external authored agency-specific blogs on usage | | | | | | |
| Agency 1 | | | | | | |
|  | AR, seasonal | 1 | .992 | .004 | 264.098 | <.001 |
|  | MA, seasonal | 1 | .934 | .015 | 60.943 | <.001 |
| Agency 2 | | | | | | |
|  | MA, seasonal | 1 | .866 | .018 | 49.358 | <.001 |
| Agency 3 | | | | | | |
|  | AR, seasonal | 1 | .999 | .003 | 377.057 | <.001 |
|  | MA, seasonal | 1 | .988 | .018 | 55.583 | <.001 |
| Agency 4 | | | | | | |
|  | AR | 1 | .917 | .048 | 18.987 | <.001 |
|  | MA | 1 | .857 | .062 | 13.815 | <.001 |
|  | AR, seasonal | 1 | .997 | .002 | 492.591 | <.001 |
|  | MA, seasonal | 1 | .965 | .012 | 77.726 | <.001 |
| Agency 5 | | | | | | |
|  | AR, seasonal | 1 | .987 | .006 | 167.695 | <.001 |
|  | MA, seasonal | 1 | .896 | .019 | 47.582 | <.001 |
| E. Temporary impact of internal authored agency-specific blogs on usage | | | | | | |
| Agency 1 | | | | | | |
|  | AR, Seasonal | 1 | .992 | .004 | 264.098 | <.001 |
|  | MA, Seasonal | 1 | .934 | .015 | 60.943 | <.001 |
| Agency 2 | | | | | | |
|  | MA, Seasonal | 1 | .866 | .018 | 49.358 | <.001 |
| Agency 3 | | | | | | |
|  | AR, Seasonal | 1 | .999 | .003 | 377.057 | <.001 |
|  | MA, Seasonal | 1 | .988 | .018 | 55.583 | <.001 |
| Agency 4 | | | | | | |
|  | AR, | 1 | .917 | .048 | 18.987 | <.001 |
|  | MA, | 1 | .857 | .062 | 13.815 | <.001 |
|  | AR, Seasonal | 1 | .997 | .002 | 492.591 | <.001 |
|  | MA, Seasonal | 1 | .965 | .012 | 77.726 | <.001 |
| Agency 5 | | | | | | |
|  | AR, Seasonal | 1 | .987 | .006 | 166.496 | <.001 |
|  | MA, seasonal | 1 | .896 | .019 | 47.411 | <.001 |
| F. Sustained impact of internal authored agency-specific blogs on usage | | | | | | |
| Agency 1 | | | | | | |
|  | AR, Seasonal | 1 | .992 | .004 | 264.098 | <.001 |
|  | MA, Seasonal | 1 | .934 | .015 | 60.943 | <.001 |
| Agency 2 | | | | | | |
|  | MA, Seasonal | 1 | .866 | .018 | 49.358 | <.001 |
| Agency 3 | | | | | | |
|  | AR, Seasonal | 1 | .999 | .003 | 377.057 | <.001 |
|  | MA, Seasonal | 1 | .988 | .018 | 55.583 | <.001 |
| Agency 4 | | | | | | |
|  | AR, | 1 | .917 | .048 | 18.987 | <.001 |
|  | MA, | 1 | .857 | .062 | 13.815 | <.001 |
|  | AR, Seasonal | 1 | .997 | .002 | 492.591 | <.001 |
|  | MA, Seasonal | 1 | .965 | .012 | 77.726 | <.001 |
| Agency 5 | | | | | | |
|  | AR, Seasonal | 1 | .987 | .006 | 167.695 | <.001 |
|  | MA, seasonal | 1 | .896 | .019 | 47.582 | <.001 |
